# A Simple and Efficient Method to Cryopreserve Human Ejaculated and Testicular Spermatozoa in −80°C Freezer

**DOI:** 10.3389/fgene.2021.815270

**Published:** 2022-01-28

**Authors:** Xiaohan Wang, Fangting Lu, Shun Bai, Limin Wu, Lingli Huang, Naru Zhou, Bo Xu, Yangyang Wan, Rentao Jin, Xiaohua Jiang, Xianhong Tong

**Affiliations:** ^1^ Provincial Hospital Affiliated to Anhui Medical University, Hefei, China; ^2^ Division of Life Sciences and Medicine, Reproductive and Genetic Hospital, The First Affiliated Hospital of USTC, University of Science and Technology of China, Hefei, China

**Keywords:** human sperm, cryopreservation, −80°C freezer, testicular sperm, liquid nitrogen vapor rapid freezing

## Abstract

Human autologous sperm freezing involves ejaculated sperm, and testicular or epididymal puncture sperm freezing, and autologous sperm freezing is widely used in assisted reproductive technology. In previous studies, researchers have tried to cryopreserve sperm from mammals (rats, dogs, etc.) using a −80°C freezer and have achieved success. It is common to use liquid nitrogen vapor rapid freezing to cryopreserve human autologous sperm. However, the operation of this cooling method is complicated, and the temperature drop is unstable. In this study, we compared the quality of human ejaculation and testicular sperm after liquid nitrogen vapor rapid freezing and −80°C freezing for the first time. By analyzing sperm quality parameters of 93 ejaculated sperm and 10 testicular sperm after liquid nitrogen vapor rapid freezing and −80°C freezing, we found reactive oxygen species (ROS) of sperm of the −80°C freezer was significantly lower than liquid nitrogen vapor rapid freezing. Regression analysis showed that progressive motility, ROS, and DNA fragmentation index (DFI) in post-thaw spermatozoa were correlated with sperm progressive motility, ROS, and DFI before freezing. For the freezing method, the −80°C freezer was positively correlated with the sperm progressive motility. Among the factors of freezing time, long-term freezing was negatively correlated with sperm progressive motility and ROS. Although freezing directly at −80°C freezer had a slower temperature drop than liquid nitrogen vapor rapid freezing over the same period, the curves of the temperature drop were similar, and slight differences in the freezing point were observed. Furthermore, there were no statistically significant differences between the two methods for freezing testicular sperm. The method of direct −80°C freezing could be considered a simplified alternative to vapor freezing for short-term human sperm storage. It could be used for cryopreservation of autologous sperm (especially testicular sperm) by *in vitro* fertilization centers.

Clinical Trial Registration: (website), identifier (ChiCTR2100050190).

## Introduction

Human sperm cryopreservation has been widely used for human reproduction. Autologous sperm freezing is the general method applied *in vitro* fertilization (IVF) laboratories in a variety of circumstances ranging from fertility preservation for cancer patients to the clinical management of male infertility (Trottmann et al., 2007).

The first attempt to freeze semen dates can be traced back to 1776 when Abbot et al*.* (Royere, et al., 1996) reported that snow could store sperm by cooling. Advances in cryobiology seen over the past decades and cryoprotective medium, in particular, have significantly aided sperm freezing-resuscitation technology (Sherman, 1973). A variety of freezing methods have been discovered with the development of cryopreservation technology: slow freezing, liquid nitrogen vapor rapid freezing, and vitrification (Tao et al., 2020). Currently, the mainstream method for semen freezing or freezing a small number of spermatozoa samples is liquid nitrogen vapor rapid freezing (vapor rapid freezing) (Huang et al., 2020). The standard cryopreservation method normally involves freezing human sperm in liquid nitrogen vapor to −80°C and then stored in liquid nitrogen. Previous reports had shown that samples are placed at 1–10 cm above the liquid nitrogen surface for 10–30 min, followed by storage in liquid nitrogen (Gwatkin, 1991; Le et al., 2019). Vapor rapid freezing cannot control the rate of temperature drop caused by the volatilization of liquid nitrogen (Di Santo et al., 2012). Another limitation of liquid nitrogen vapor rapid freezing is that it cannot cool many semen samples simultaneously.

Freezing semen samples by directly placing them in a −80°C freezer is the most practical means because a −80°C freezer is readily available. A few researchers have reported that several mammalian spermatozoa can be successfully frozen by direct placement in a −80°C freezer. For example, Marcello Raspa et al. (2017), Raspa et al. (2018a), Raspa et al. (2018b), reported that mouse spermatozoa can be frozen, transported, shared, and stored at −80°C for a long time without a significant loss of viability. Pezo et al. (2017) demonstrated that semen freezing and storage using a −80°C ultra-freezer is an effective technique for the long-term preservation of canine spermatozoa. Parkes. (1945) indicated that human sperm cryopreservation without cryoprotectants at −79°C offered an advantage over storage at −196°C. In addition, Liu et al. (2016) concluded that storage of neat semen samples at −80°C caused milder damage to sperm DNA than storage at −196°C mixed with cryoprotectants. In 2012, Sanchez et al. (2012) concluded that there were no significant differences in sperm progressive motility, the integrity of mitochondrial membrane potential (MMP) or DNA fragmentation for vitrified swim-up human sperm either at −196°C under liquid nitrogen or at -80°C. However, Vaz et al. (2018) found storage of human sperm at −80°C freezer up to 96 hours was detrimental to sperm viability. Therefore, the impact of −80°C freezer on human sperm needs to be further explored.

The cryopreservation of limited spermatozoa from men afflicted with nonobstructive azoospermia using testicular sperm aspiration (TESA) can avoid repeated surgery and promote the preservation of fertility (Miller et al., 2017). Current research on the freezing of testicular or epididymal sperm is mainly focused on single sperm freezing. In 1997, Cohen et al. (1997) first described a novel cryopreservation technique for single sperm using an empty zona pellucida (ZP). Various single sperm cryopreservation carriers have been proposed in the last 20 years, including ZPs (Hsieh et al., 2000; Just et al., 2004), cryoloops (Desai et al., 2004), culture dishes (Sereni et al., 2008), cell sleepers (Coetzee et al., 2016), cryotops (Endo et al., 2012), and novel sperm vitrification devices (Berkovitz et al., 2018). Each cryopreservation method has limitations; in particular, the usage of these carriers requires experienced technicians to select and capture single sperm, which always results in very few available sperm after this process. Therefore, frozen testicular sperm with single sperm has not become widespread, and an ideal container that can be universally used needs to be developed.

To explore whether freezing sperm at −80°C freezer is feasible for human ejaculate and testicular spermatozoa, we evaluated the effects of freezing sperm at −80°C freezer and vapor rapid freezing techniques on sperm quality, and simplified the procedure and equipment for freezing human ejaculate and testicular spermatozoa.

## Materials and Methods

The procedures in this study were approved by the Ethical Committee of The First Affiliated Hospital of the University of Science and Technology of China (2021-KY-040), and informed consent was obtained from all subjects.

### Source of Sperm Samples

The study included 93 ejaculated sperm from normozoospermic men who sought fertility evaluation at the Reproductive Center of The First Affiliated Hospital of University of Science and Technology of China between March 2021 and June 2021. And ten testicular tissue from patients who were diagnosed with obstructive azoospermia. Based on medical history and seminal examination findings, patients with vasectomy, varicocele, cryptorchidism, or genital infection were excluded from the analysis. Patients receiving any medication or antioxidant supplementation in 3 months before the study were also excluded.

### Experimental Design

**FIGURE 1:**
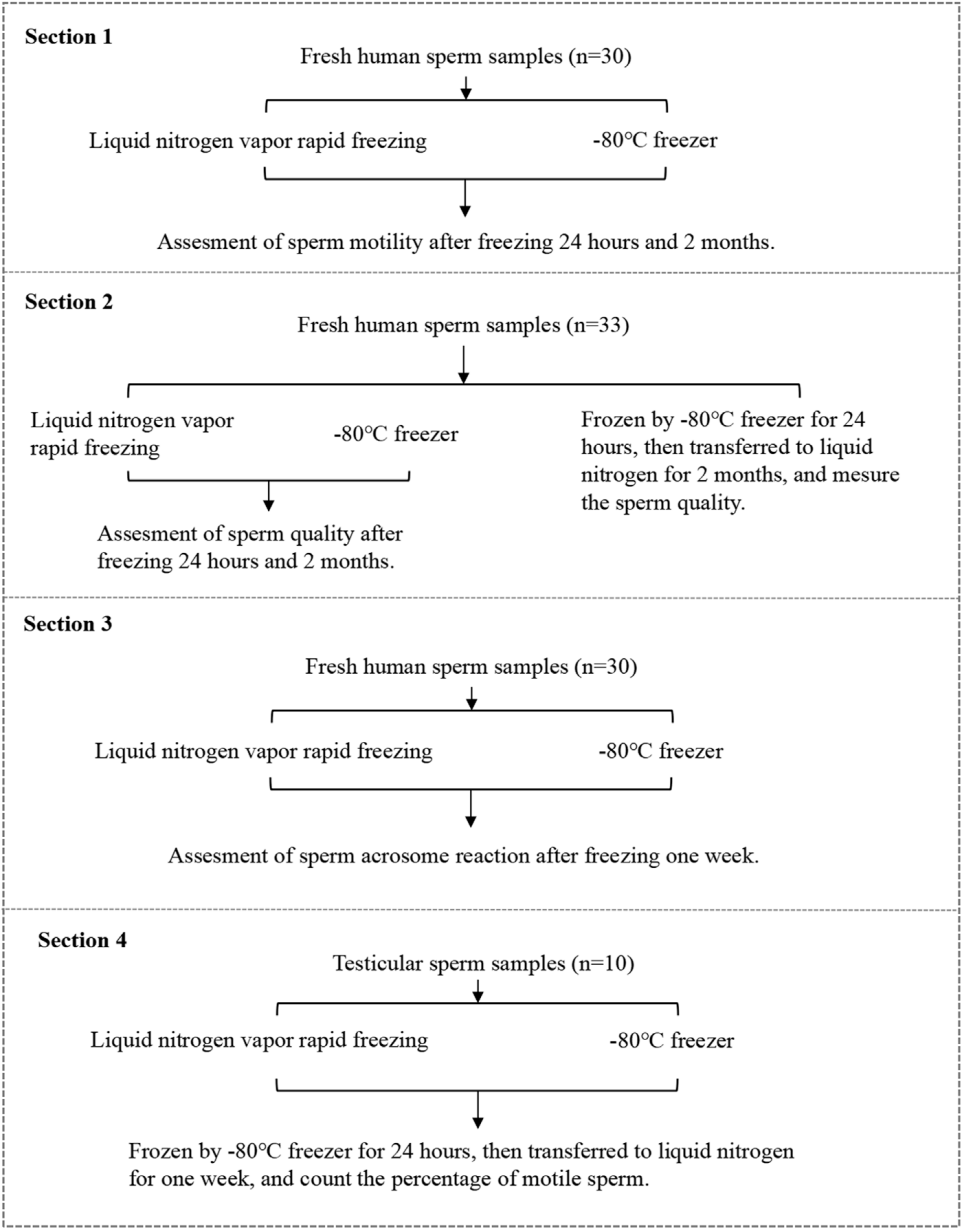
Diagrammatic representation of the experimental design.

A diagram of the experimental design is shown in Figure 1. The experiments were split into four sections.

#### Section 1

Thirty ejaculated sperm from normozoospermic men were analyzed for progressive motility and viability by vapor rapid freezing or direct −80°C freezing. The sperm samples were thawed and sperm parameters were measured 24 h (short-term) or 2 months (long-term) later.

#### Section 2

Thirty-three ejaculated sperm from normozoospermic men were analyzed for sperm quality by vapor rapid freezing or direct −80°C freezing, kept for 24 h in a −80°C freezer and then moved into liquid nitrogen. The sperm samples were thawed and sperm parameters were measured 24 h (short-term) or 2 months (long-term) later.

Overall, there were five groups in section 1 and section 2 (Ⅰ) frozen by vapor rapid freezing for short-term (short vapor group); (Ⅱ) frozen by vapor rapid freezing for long-term (long vapor group); (Ⅲ) frozen in -80°C freezers for short-term (short freezer group); (Ⅳ) frozen in −80°C freezers for long-term (long freezer group); (Ⅴ) frozen in −80°C freezers for 24 h and then immersed in liquid nitrogen for 2 months (liquid nitrogen group).

#### Section 3

Thirty ejaculated sperm from normozoospermic men were analyzed for acrosome reaction after a week by vapor rapid freezing and direct −80°C freezing.

#### Section 4

Vapor rapid freezing and direct −80°C freezing were used to freeze 10 testicular sperm for a week. This section was designed to compare the percentage of motile sperm between vapor rapid freezing and freezing at −80°C.

### Method of −80°C Freezing

The liquefied ejaculated semen was diluted with sperm freezing media (ORIGIO, Måløv, Denmark) (ratio 1:1), and 1 ml of the suspension was pipetted into a Nunc cryotube vial (1.8 ml; catalog number: 375418, Thermo Fisher Scientific, Jiangsu, China), which was kept at room temperature (RT) for 10 min and subsequently placed in a horizontal position in the freezer. Some samples were transferred to liquid nitrogen 24 h later. The purpose of placing the container in a horizontal position was to minimize the heat difference between the two ends during freezing (Di Santo et al., 2012).

### Method of Liquid Nitrogen Vapor Rapid Freezing

After 1 ml of suspension was pipetted into a 1.8 ml aseptic cryotube, the samples were kept at RT for 10 min and subsequently placed horizontally 8–10 cm above the liquid nitrogen surface. Fifteen minutes later, it was submerged in liquid nitrogen and stored at −196°C in a tank full of liquid nitrogen for 24 h or 2 months.

### Method of Ejaculated Sperm Thawing

Thawing was performed as described previously for a specific sperm freezing medium with some modifications (ORIGIO, Måløv, Denmark). The cryotube was removed from the liquid nitrogen or −80°C freezer and submerged in warm water (37°C) for 10 min. Post-thaw sperm progressive motility, viability, reactive oxygen species (ROS), MMP, DNA fragmentation index (DFI), and high DNA stainability (HDS) were analyzed.

### Cryopreserved Spermatozoa Collected by TESA

After removing the seminiferous tubules from the testicles, the sample was placed in a dish with 5% 3-(N-Morpholino) propanesulphonic acid with gentamicin (G-MOPS^TM^, Vitrolife, Sweden) medium and minced with a needle connected to a 1 ml syringe under a dissecting microscope. Then, the sample was examined under an inverted microscope (Olympus, Tokyo, Japan) at 400× magnification. Once spermatozoa (motile or not motile) were observed in the dish, the suspension was mixed by pipetting and transferred to a tube with SpermRinse (Vitrolife, Sweden), and then the tube was placed upright for 5 min. After removing the large sludge at the bottom using a straw, the supernatant was centrifuged at 250 g for 5 min, and approximately 0.5 ml of sediment was reserved. An equal volume of sperm freezing medium (ORIGIO, Måløv, Denmark) was added dropwise onto the sediment, and the solution was carefully mixed after each addition, followed by incubation at RT for 10 min. Each sample was divided into two tubes, which were frozen using vapor rapid freezing or −80°C freezers. After 24 h, the samples frozen in the -80°C freezer were transferred to liquid nitrogen.

### Thawing of Spermatozoa Collected by TESA

The sperm obtained from the testicular puncture and frozen for 1 week were thawed in a 37°C-water bath for 10 min. Then, we used SpermRinse to remove the cryoprotectant in the semen sample and centrifuged it to remove the supernatant. After adding pentoxifylline to the sample for sperm activation, the sample was filled into the sperm counting pool, and the number of sperm and the percentage of motile sperm were counted under a microscope.

### Temperature Curve Assessment

The temperature changes during freezing were determined using a portable, multiuse industrial data logger (OM-CP-OCTPRO, Omega Engineering, United States). The temperature sensor uses a thermocouple K matched with the industrial data logger.

### Routine Semen Analyses

Routine semen analyses were performed using computer-assisted semen analysis (CASA) to determine progressive motility. We analyzed a minimum of six fields of view per chamber and, at least 200 sperm were evaluated in each chamber according to World Health Organization (WHO) guidelines (WHO, 2010).

Sperm viability was evaluated by eosin-nigrosine staining (Ankebio, China). At least 200 spermatozoa were analyzed with an optical microscope (magnification 1000×). Sperm with red heads were considered nonviable (membrane-damaged), whereas sperm showing no color were considered alive (membrane-intact) (Rarani et al., 2019).

### The Sperm Chromatin Structure Assay

Sperm Chromatin Structure Assay (SCSA) was measured by flow cytometry according to the protocol based on Evenson et al. (1980). A commercial kit (Cellpro, China) was used for the evaluation of SCSA. Damaged chromatin in the sperm nucleus after acid treatment forms a single chain and emits red or orange fluorescence upon binding the dye acridine orange; normal sperm chromatin in the nucleus maintains the integrity of the double-stranded structure after acid treatment and emits green fluorescence when combined with acridine orange. At least 5,000 cells were counted per sample. The SCSA parameters include DFI defined as the percentage of denatured sperm DNA and HDS defined as the percentage of spermatozoa with abnormally high DNA stainability.

### Reactive Oxygen Species Assessment

Reactive oxygen species (ROS) were determined using a Sperm Reactive Oxygen Species Detection Kit (Ankebio, China), flow cytometry was used for detection, and each sample contained at least 5,000 cells (Mahfouz et al., 2009).

### Mitochondrial Membrane Potential Assessment

To measure the mitochondrial membrane potential (MMP) of the sperm, a sperm mitochondrial staining kit (JC-1 fluorescent staining method, Ankebio, China) was used. The MMP in sperm cells can be labeled with fluorescent dyes. The fluorescent probes gather in the mitochondria and emit red fluorescence when there is high MMP. At least 5,000 cells were counted per sample.

### Sperm Acrosome Reaction Assessment Statistical Analysis

According to the WHO guidelines, the acrosome reaction (AR) was assessed by Fluorescein isothiocyanate-Pisum sativum agglutinin (FITC-PSA, Sigma-Aldrich, St. Louis, America) staining. After rinsing sperm with phosphate buffer saline, sperm were fixed with 4% (w/v) paraformaldehyde or 10 min, mounted on slides, then air-dried and incubated 2 h in the dark at 4°C with 25 mg/L FITC-PSA. Sperm were washed with PBS and examined by fluorescence Nikon Eclipse 80i microscopy (n b 200 sperm/ sample) (Nikon Inc., Tokyo, Japan).

The results were analyzed by using the program Statistical Product and Service Solutions (SPSS) Statistics 23.0 (SPSS Inc., Chicago, IL, United States). Data are expressed as the means ± standard deviation (SD). The comparison between the two groups was performed by paired-samples *t*-test. The percentage of motile sperm was expressed as proportion, and *p*-value was derived from the chi-square test. Regression analysis was used to compare the correlation between sperm parameters. A statistical value of *p* < 0.05 was considered statistically significant.

## Results

### Characteristics of the Study Population

A total of ninety-three specimens from patients attending the reproductive center were enrolled. The characteristics of the study population are shown in Table 1. Among the 93 participants, the mean age was 32.23 ± 4.96 years and the mean semen volume was 3.85 ± 1.31 ml. The mean of sperm parameters, such as sperm concentration, viability, and progressive motility, were all above the reference established by the WHO (2010).

**TABLE 1:** Characteristics and sperm parameters in patients from sections 1, 2 and 3.

Characteristics	Section 1 (*n* = 30)	Section 2 (*n* = 33)	Section 3 (*n* = 30)	Total (*n* = 93)
Age (year)	30.80 ± 3.99	32.82 ± 4.87	33.07 ± 6.03	32.23 ± 4.96
Sperm volume (ml)	4.08 ± 1.17	3.48 ± 1.29	4.00 ± 1.47	3.85 ± 1.31
Sperm concentration (million/mL)	70.94 ± 47.23	61.08 ± 37.85	87.62 ± 36.40	73.21 ± 40.49
Progressive motility (%)	36.08 ± 9.40	36.28 ± 12.13	44.93 ± 7.16	39.10 ± 9.56
Viability (%)	73.95 ± 13.59	71.01 ± 14.12	68.02 ± 9.51	70.99 ± 12.41
ROS (%)	—	10.25 ± 9.38	—	—
MMP (%)	—	88.81 ± 14.18	—	—
DFI (%)	—	9.90 ± 4.22	—	—
HDS (%)	—	6.75 ± 2.75	—	—

ROS, reactive oxygen species; MMP, mitochondrial membrane potential; DFI, DNA, fragmentation index; HDS, high DNA, stainable. Data were presented as mean ± SD.

### Freezing Temperature Curve of Vapor Rapid Freezing and −80°C Freezing

**FIGURE 2:**
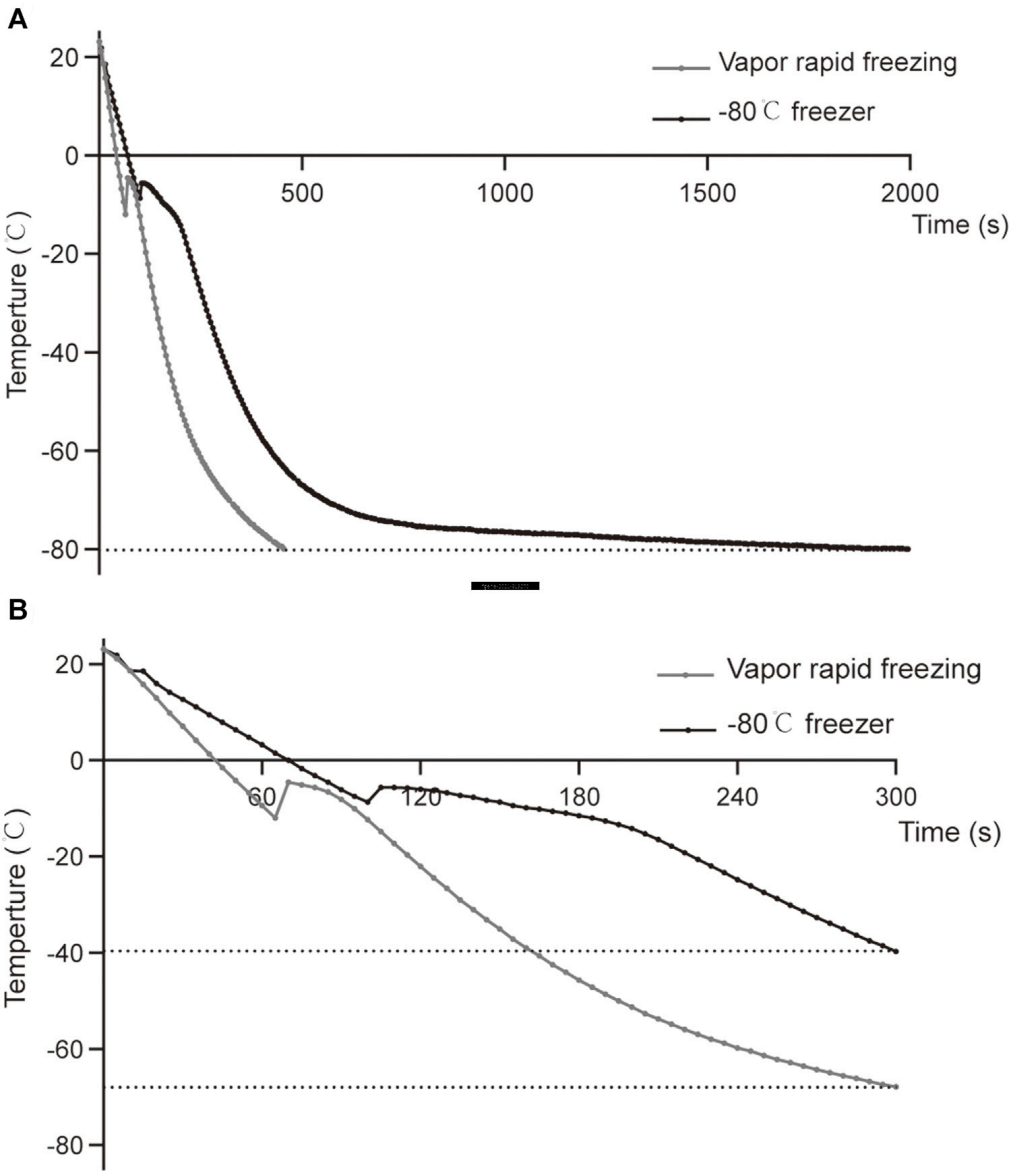
Comparison of temperature curves of vapor rapid freezing and −80°C freezer. **(A)** The temperature drop curve of sperm samples from RT to −80°C in liquid nitrogen vapor rapid freezing and −80°C freezer. **(B)** The temperature drop curve of the sperm samples in the first 300 s of liquid nitrogen vapor rapid freezing and −80°C freezer.

The sample was placed in liquid nitrogen vapor for 5 min, and the cooling rate was −18°C/min down from RT to −65°C. The drop from RT to −80°C took approximately 410 s (Figure 2, Supplementary Table 1). In the −80°C freezer, the sample was placed in liquid nitrogen vapor for 5 min, the cooling rate was approximately -12°C/min down from RT to −40°C, and the drop from RT to −80°C took approximately 1990 s (Figure 2, Supplementary Table 1). Although freezing at −80°C has a slower temperature drop than vapor rapid freezing during the same period, the curves of the temperature drop are similar between the two methods. Notably, slight differences in the temperature were observed at the freezing point (Figure 2B, Supplementary Table 1).

### Effect of Different Cryopreservation Methods and Different Storage Times on Sperm Quality

As shown in Table 2, no significant difference was observed in progressive motility and viability across the different freezing methods (*p* > 0.05).

**TABLE 2:** Comparison of sperm motility and viability between liquid nitrogen vapor rapid freezing and −80°C freezer.

Groups	Progressive motility (%)	Viability (%)
Short vapor group (*n* = 63)	14.67 ± 4.99	44.33 ± 9.92
Short freezer group (*n* = 63)	15.53 ± 5.40	46.02 ± 10.84
*p-v*alue	0.052	0.056
Long vapor group (*n* = 63)	12.97 ± 4.94	41.02 ± 9.75
Long freezer group (*n* = 63)	12.87 ± 4.84	40.77 ± 9.52
*p-v*alue	0.793	0.741
Long vapor group (*n* = 33)	11.77 ± 5.19	38.69 ± 10.18
Liquid nitrogen group (*n* = 33)	11.10 ± 4.86	37.43 ± 8.67
*p-v*alue	0.091	0.120

Short vapor group: frozen by vapor rapid freezing for 24 h; Short freezer group: frozen by −80°C freezer for 24 h; Long vapor group: frozen by vapor rapid freezing for 2 months; Long freezer group: frozen by −80°C freezer for 2 months; Liquid nitrogen group: frozen by −80°C freezers for 24 h and then immersing it in liquid nitrogen for 2 months; *p-v*alue was derived from paired *t*-test. Data were presented as mean ± SD.

The ROS of sperm of the freezer group was significantly lower than that of the vapor group (Table 3). However, the HDS of sperm in the long freezer group was significantly higher than that of sperm in the long vapor group (*p* < 0.05). There was no significant difference in the other parameters between the different methods (*p* > 0.05).

**TABLE 3:** Comparison of sperm function between liquid nitrogen vapor rapid freezing and −80°C freezer.

Groups	ROS (%)	MMP (%)	DFI (%)	HDS (%)
Short vapor group (*n* = 33)	9.53 ± 8.47	84.95 ± 17.73	9.41 ± 4.62	7.87 ± 2.95
Short freezer group (*n* = 33)	7.45 ± 7.14	87.04 ± 14.68	9.45 ± 4.47	8.20 ± 2.87
*p-v*alue^a^	0.036	0.611	0.805	0.133
Long vapor group (*n* = 33)	12.94 ± 9.46	75.91 ± 19.39	9.53 ± 4.74	8.46 ± 2.90
Long freezer group (*n* = 33)	11.64 ± 8.13	75.30 ± 21.19	9.72 ± 5.30	9.10 ± 2.88
Liquid nitrogen group (*n* = 33)	12.48 ± 9.35	77.75 ± 20.18	9.47 ± 4.44	8.65 ± 2.63
*p-v*alue^b^	0.022	0.713	0.634	0.017
*p-v*alue^c^	0.655	0.339	0.791	0.343

Short vapor group: frozen by vapor rapid freezing for 24 h; Short freezer group: frozen by −80°C freezer for 24 h; Long vapor group: frozen by vapor rapid freezing for 2 months; Long freezer group: frozen by −80°C freezer for 2 months; Liquid nitrogen group: frozen by −80°C freezers for 24 h and then immersing it in liquid nitrogen for 2 months.

a *p-v*alue of short freezer group versus short vapor group (paired *t*-test).

b *p-v*alue of long freezer group versus long vapor group (paired *t*-test).

c *p-v*alue of liquid nitrogen group versus long vapor group (paired *t*-test). Data were presented as mean ± SD.

**FIGURE 3:**
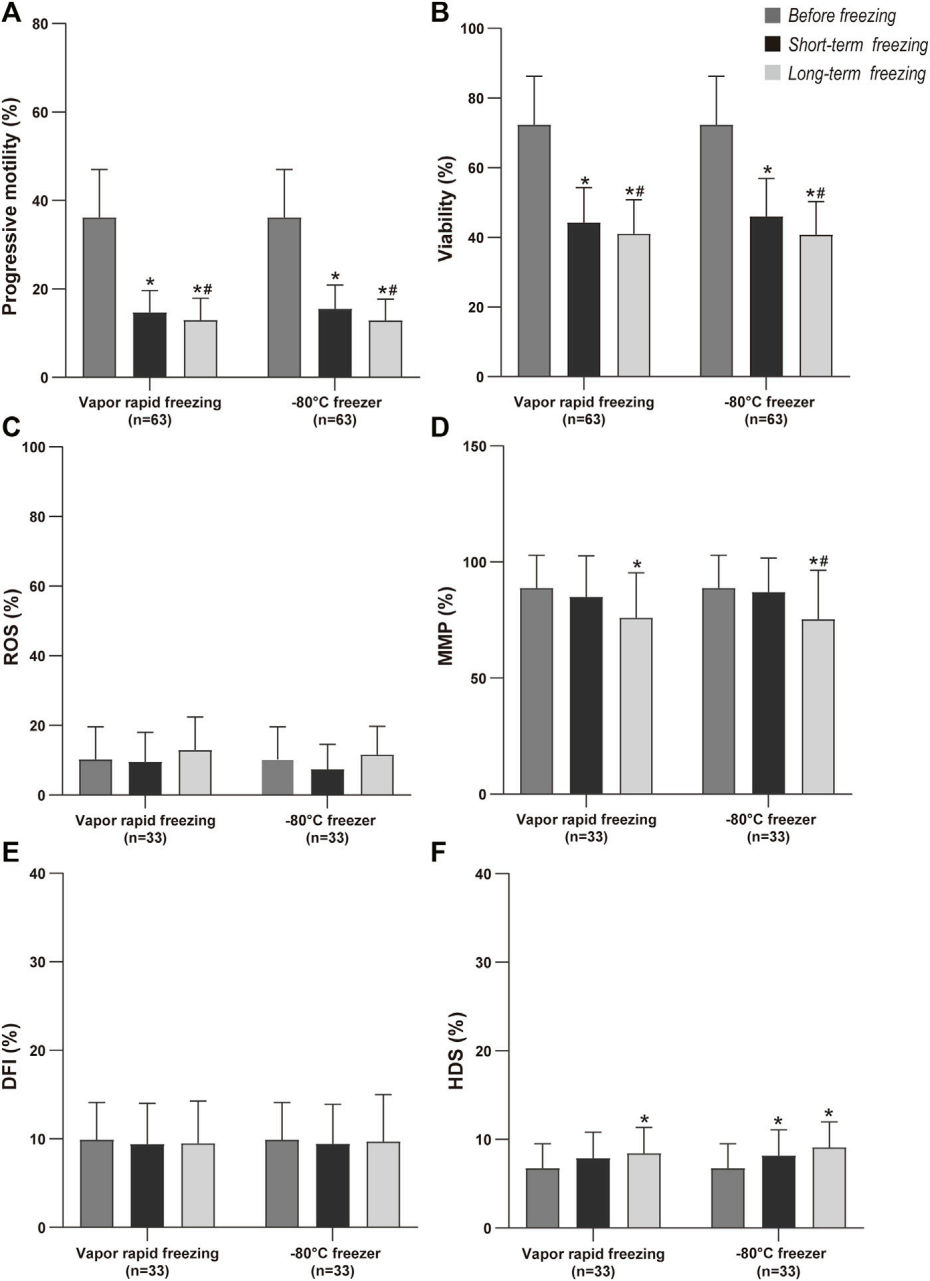
Comparison of sperm quality by cryopreservation time before freezing, short term and long-term freezing. Values are mean and bar are SD; *p-value < 0.05 compare with before freezing (paired t-test); ^#^p-value < 0.05 compare with short-term freezing (paired t-test).

We compared the state of the sperm after freezing and found that progressive motility and viability were significantly decreased (Figures 3A,B). Specifically, compared with short-term freezing, long-term freezing significantly decreased sperm motility. There was no significant difference in ROS before and after freezing (Figure 3C), while long-term freezing decreased MMP (Figure 3D). Moreover, long-term storage of sperm had a similar DFI with short-term storage (Figure 3E). In addition, freezing also resulted in a higher HDS in the sperm (Figure 3F).

### Effect of Different Cryopreservation Methods on Sperm Acrosome Reaction

**FIGURE 4:**
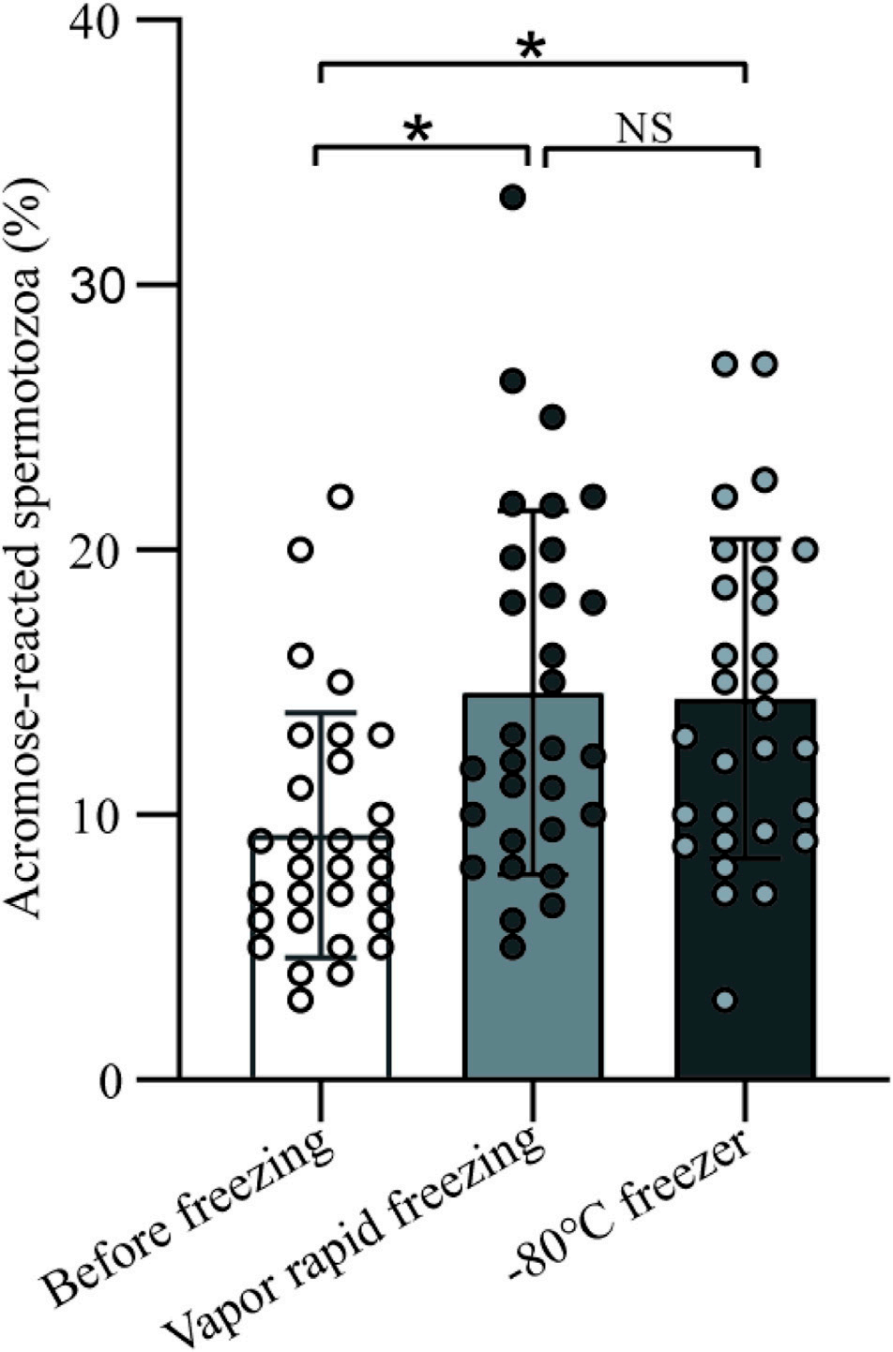
The impact on sperm acrosome of vapor rapid freezing and −80°C freezer. Sperm treated by freezing had significant higher acrosome reaction than before freezing (*p*-value < 0.05). There was no significant difference in the acrosome reaction between vapor rapid freezing and −80°C freezer (*p*-value > 0.05).

A total of 30 men were included and significant differences (9.23 ± 4.61 vs. 14.62 ± 6.86 vs. 14.38 ± 6.05, *p* < 0.05) were observed for acrosome reaction (AR) within before and freeze-thawed spermatozoa (Figure 4). To be noted, there was no significant difference in the AR between vapor rapid freezing and −80°C freezer (14.62 ± 6.86 vs. 14.38 ± 6.05, *p* > 0.05).

### Linear Regression Between Sperm Quality After Freeze-Thawing and Sperm Parameters

In regression analysis, we found that progressive motility in post-thaw spermatozoa was positively correlated with both progressive motility before freezing and the direct −80°C freezing methods but negatively correlated with ROS, DFI before freezing, and storage time (*p* < 0.05, Table 4). ROS in post-thaw spermatozoa was positively correlated with ROS before freezing and storage time (*p* < 0.05). DFI in post-thaw spermatozoa was positively correlated with DFI before freezing but negatively correlated with ROS before freezing (*p* < 0.05).

**TABLE 4:** Results from the regression analyses after freezing sperm quality.

Parameters	Progressive motility after freezing		ROS after freezing		DFI after freezing	
	β (95%CI)	*p-v*alue	β (95%CI)	*p-v*alue	β (95%CI)	*p-v*alue
Progressive motility before freezing	0.30 (0.25–0.35)	<0.001	0.06 (-0.06–0.18)	0.331	−0.14 (−0.20–0.08)	<0.001
ROS before freezing	−0.13 (−0.23–0.01)	0.010	0.19 (0.03–0.34)	0.021	−0.05 (−0.13–0.04)	0.314
DFI before freezing	−0.44 (−0.65–0.23)	<0.001	−0.06 (−0.47–0.30)	0.577	0.93 (0.82–1.04)	<0.001
Time						
Short-term	Ref	Ref	Ref	Ref	Ref	Ref
Long-term	−2.49 (−3.84–1.14)	<0.001	3.80 (1.02–6.59)	0.008	0.19 (−0.74–1.12)	0.688
Method						
Vapor rapid freezing	Ref	Ref	Ref	Ref	Ref	Ref
−80°C freezer	1.64 (0.29–2.99)	0.018	−1.69 (−4.48–1.09)	0.231	0.12 (−0.81–1.05)	0.804

β, regression analyses coefficients; 95% CI, 95% confidence interval.

### Motile Sperm Percentage of Testicular Sperm Samples After Cryopreservation With Vapor Rapid Freezing and −80°C Freezer

To analyze the outcomes of freeze-thawed spermatozoa collected by TESA, we compared the parameters of sperm conserved by vapor rapid freezing and stored at −80°C (Table 5). We counted a total of 62 motile sperm, accounting for 16.89% of the total sperm, when vapor rapid freezing was used. A total of 65 motile sperm, accounting for 17.02% of the total sperm, were counted when freezing at −80°C. There was no statistically significant difference between the two methods for freezing testicular sperm (*p* = 0.965).

**TABLE 5:** Motile sperm percentage of testicular sperm samples after cryopreservation with vapor rapid freezing and -80°C freezer.

Sample number	Percentage of motile sperm (%)		*p-v*alue
	Vapor rapid freezing	-80°C freezer	
1	7/53 (13.21)	6/48 (12.50)	-
2	7/38 (18.42)	4/24 (16.67)	-
3	4/29 (13.79)	6/31 (19.35)	-
4	3/33 (9.09)	4/38 (10.52)	-
5	13/57 (22.81)	10/48 (20.83)	-
6	4/24 (16.67)	6/37 (16.21)	-
7	3/19 (15.79)	3/18 (16.67)	-
8	15/63 (23.81)	17/72 (23.61)	-
9	2/20 (10.00)	3/27 (11.11)	-
10	4/31 (12.90)	6/39 (15.38)	-
Total	62/367 (16.89)	65/382 (17.02)	0.965

*p-v*alue was derived from chi-square test.

## Discussion

This study compared the effects of a −80°C freezer and vapor rapid freezing on sperm quality, and we found that there was no significant difference in progressive motility and viability across the different freezing methods. Correlation analysis showed that progressive motility, ROS, and DFI in the post-thaw spermatozoa were correlated with sperm characteristics before freezing, methods and storage time.

Some studies have reported that freezing-thawing decreases sperm quality and function (Satirapod et al., 2012; Lusignan et al., 2018; Le et al., 2019). For example, proteomic analysis of sperm showed significant changes in proteins related to motility, viability, and acrosomal integrity of sperm compared with the fresh state (Wang et al., 2014). It has also been suggested that rapid changes in intracellular ice crystal formation and osmolarity during the cryopreservation process may lead to changes in the carbohydrate composition and the membrane proteins, which can disrupt membrane structures and reduce sperm viability (Pedersen and Lebech, 1971). The production of ROS and lower antioxidant enzyme activity in sperm induce apoptotic pathways, which can lead to a reduction in sperm viability (Di Santo et al., 2012). In our study, we found the same results as previous research that showed that after freezing-thawing, sperm viability and progressive motility were both decreased significantly. ROS increased and MMP continued to decrease as the freezing time extended. However, compared with vapor rapid freezing, there was no significant difference in progressive motility, viability, MMP or DFI of sperm frozen in a −80°C freezer. Previously, Rahana et al. (2011) also reported similar results that there was no significant difference in human sperm motility and DFI between -85°C and conventional liquid nitrogen cryopreservation. Previous studies observed a decrease in AR after the freezing-thawing process (Gomez-Torres et al., 2017; Rahiminia et al., 2017). Here we also observed that the acrosome status was vulnerable to the freezing-thawing process, but the percentages of AR decrease for different cryopreservation methods was similar, which further confirmed the efficiency of −80°C freezer.

In regression analysis, compared with vapor rapid freezing, −80°C freezing was positively correlated with sperm progressive motility. Our study also indicated that freezing in a −80°C freezer resulted in lower ROS than vapor rapid freezing, which confirmed that a slow temperature drop would result in lower ROS damage. During freezing, the cooling rate of vapor rapid freezing was quicker than that of the −80°C freezer, and thus vapor rapid freezing took less time to reach the freezing point and had a lower freezing point temperature than the −80°C freezer (Supplementary Table 1). In the freezing process, water tends to chill beyond its freezing point without forming ice, which is known as supercooling. Studies have confirmed that super-cooling can cause damages of sperm in mice and human (Check et al., 1995; Mazur and Koshimoto, 2002). Compared with vapor rapid freezing, the samples frozen in a −80°C freezer have a higher freezing point that is closer to −6°C (Supplementary Table 1). The WHO (2010) first recommended that a human sperm freezing program decline from RT (22–25°C) to −6°C, and we speculated that −6°C was the freezing point of semen with cryoprotectant. Therefore, samples in a −80°C freezer were less supercooled than vapor rapid freezing, which may result in less damage. Notably, the HDS of sperm frozen in a −80°C freezer for a long time was higher than that in liquid nitrogen. Compared with vapor rapid freezing, the −80°C freezer has a more stable and slower cooling rate and can meet the demand for sperm freezing so that the freezing step is simplified. Thus, although liquid nitrogen storage is irreplaceable and is still the first choice for long-term sperm cryopreservation at a low temperature of −196°C, a −80°C freezer can be an alternative method for short-term sperm storage.

In this study, we used 1.8 ml cryotubes and a −80°C freezer to simplify the steps of freezing testicular sperm. We have proven that −80°C freezers and vapor rapid freezing have similar effects on testicular sperm. Previously, the method of freezing single sperm captured under a microscope using intracytoplasmic sperm injection (ICSI) pipettes equipped with a micromanipulator is widely used for testicular sperm (Coetzee et al., 2016). However, more sperm can be obtained after freezing all the testicular sperm in a freezing tube and a −80°C freezer compared with the freezing of single sperm. Using cryotubes will provide enough sperm after freezing-thawing, and it is better to select motile sperm for ICSI. Furthermore, the efficiency of single sperm freezing is low and cannot meet the requirements of storing numerous samples. The freezing of testicular sperm in a −80°C freezer in a freezer tube ensures that all sperm in the testicular tissue are captured, it only takes a short time and simplifies the process, and several samples can be processed simultaneously.

The present study was based on a limited sample size and normal semen meeting the WHO standard, and further studies of −80°C freezers in clinical applications with sperm samples from oligo-astheno-teratozoospermia are warranted. This was a preliminary study to improve the freezing of testicular sperm, and it was necessary to freeze tremendous testicular sperm samples to avoid instability. *In vitro* fertilization tests, follow-up embryo development, and implantation are also the focus of future research.

In conclusion, this study demonstrated that short-term storage of sperm at −80°C freezer could be a viable alternative to liquid nitrogen vapor rapid freezing at −196°C due to their comparable post-thaw results and lower ROS. During long-term freezing, the −80°C freezer is expected to be a cooling process that can provide an option except for liquid nitrogen vapor rapid freezing. An improved sperm freezing process was also preliminarily explored in this study. Additional study is necessary to confirm the clinical value of the freezing testicular sperm method.

## Data Availability

The raw data supporting the conclusion of this article will be made available by the authors, without undue reservation.

## Abbreviations

DFI, DNA fragmentation index; FITC-PSA, fluorescein isothiocyanate-pisum sativum agglutinin; HDS, high DNA stainability; ICSI, intracytoplasmic sperm injection; IVF, *in vitro* fertilization; MMP, mitochondrial membrane potential; RT, room temperature; ROS, reactive oxygen species; SCSA, the sperm chromatin structure assay; CASA, computer-assisted semen analysis; SPSS, statistical product and service solutions; SD, standard deviations; TESA, testicular sperm aspiration; ZP, zona pellucida, AR, acrosome reaction.
