# Supplementary material for: A Simple and Efficient Method to Cryopreserve Human Ejaculated and Testicular Spermatozoa in −80°C Freezer
Source: Front Genet. 2022 Jan 28;12:815270. doi: 10.3389/fgene.2021.815270 (PMC8831890; doi:10.3389/fgene.2021.815270)
Supplement: Supplementary file 1 [file Table1.doc]

## Supplementary table

**Supplementary table 1** Comparison temperature curve of vapor rapid freezing and -80℃ freezer.

|  | Initial temperature | Freezing point | Cooling rate | Time required to -80 °C |
| --- | --- | --- | --- | --- |
| Vapor rapid freezing | 23.1°C | -12°C | 32.4°C min^-1^ | 410 seconds |
| -80℃ freezer | 23.1°C | -8.7°C | 19.1°C min^-1^ | 1990 seconds |

Initial temperature: room temperature; Freezing point: the temperature at which the sample changes from liquid to solid; Cooling rate: the rate of sample from initial temperature to freezing point; Time required to -80 °C: the time required for the sample from initial temperature to -80°C.
